# ACTIVATING THE NEXT GENERATION OF GERONTOLOGY ENTHUSIASTS... ONE SEMESTER AT A TIME

**DOI:** 10.1093/geroni/igad104.3161

**Published:** 2023-12-21

**Authors:** Joanna Culligan, Ila Schepisi

**Affiliations:** Virginia Tech, Blacksburg, Virginia, United States; Virginia Tech, Blacksburg, Virginia, United States

## Abstract

At the Engagement Center for Creative Aging at Virginia Tech, we are activating the next generation of gerontology enthusiasts while breaking down stigmas associated with aging and cognitive change. Each semester, we have an opportunity to engage with the undergraduate and graduate student population here on campus through field work, volunteerism and student employment. Here, students engage with adults in our community through a wide range of programs to support aging from community clubs, caregiver wellness and our adult day services program. This paper focuses on Reframing Aging with students who engage in field work at our adult day services program through the Department of Human Development and Family Science at Virginia Tech through strategic application of active learning pedagogical techniques including lecture, class discussion, experiential activities and application exercises. In addition to classroom components, students participate in service delivery at our center. At the beginning of each semester, students are asked (using an anonymous online polling tool), “What words do you associate with older adults” and “… older adults with dementia.” The most common words that have appeared, semester after semester, are: stubborn, inflexible, angry, sad, decline, struggling, forgetful and confused. At the end of the semester, sixteen-weeks later, students are asked the same questions. The most common words associated with older adults and those experiencing dementia at the end of the semester are: interesting, unique, wise, lovely, welcoming, insightful, experienced, helpful, normal, honest and human. In one semester, we are helping to Reframe Aging with this approach.

